# The Characterization of Ground Raspberry Seeds and the Physiological Response to Supplementation in Hypertensive and Normotensive Rats

**DOI:** 10.3390/nu12061630

**Published:** 2020-06-01

**Authors:** Michał Majewski, Ewa Kucharczyk, Roman Kaliszan, Michał Markuszewski, Bartosz Fotschki, Jerzy Juśkiewicz, Małgorzata Borkowska-Sztachańska, Katarzyna Ognik

**Affiliations:** 1 Department of Pharmacology and Toxicology, Faculty of Medicine, UWM, 10-082 Olsztyn, Poland; ewa.kucharczyk@onet.pl; 2 Department of Biopharmaceutics and Pharmacodynamics, Medical University of Gdansk, Hallera 107, 80-416 Gdansk, Poland; roman.kaliszan@gumed.edu.pl (R.K.); markusz@gumed.edu.pl (M.M.); 3 Division of Food Science, Institute of Animal Reproduction and Food Research of the Polish Academy of Sciences, 10-748 Olsztyn, Poland; b.fotschki@pan.olsztyn.pl (B.F.); j.juskiewicz@pan.olsztyn.pl (J.J.); 4 Department of Psychiatry, Faculty of Medicine, UWM, 10-228 Olsztyn, Poland; m.borkowska@wzlp.pl; 5 Department of Biochemistry and Toxicology, Faculty of Biology, Animal Sciences and Bioeconomy, University of Life Sciences, 20-950 Lublin, Poland; kasiaognik@poczta.fm

**Keywords:** COX-2, ellagic acid, ellagitannins, iNOS, lambertianin C, lambertianin D, procyanidins, raspberries, sanguiin H-6, seeds

## Abstract

This study aimed to evaluate the protective role of ground raspberry seeds (RBS) as a source of polyphenols and essential fatty acids on blood plasma enzymatic antioxidant status, lipid profile, and endothelium-intact vasodilation during physiological and pathological conditions. Young normotensive Wistar–Kyoto rats (WKYs) and spontaneously hypertensive rats (SHRs) at ten weeks of age were fed with either a control diet or were supplemented with added 7% RBS for six weeks (*n* = 6). The main component of RBS was dietary fiber (64%) and the main polyphenols were ellagitannins (1.2%) and flavan-3-ols (0.45%). Irrespective of the rat model, ground RBS decreased liver enzyme aspartate aminotransferase (0.9-fold) and hydrogen peroxide scavenging capacity (Catalase, 0.9-fold). In supplemented SHRs, preincubation with inducible nitric oxide synthase (iNOS) inhibitor 1400W, nonselective cyclooxygenase (COX) inhibitor indomethacin, selective COX-2 inhibitor NS-398, prostacyclin (PGI_2_) synthesis inhibitor tranylcypromine (TCP), thromboxane receptor (TP) antagonist SQ-29548, thromboxane synthesis inhibitor furegrelate, and 20-HETE synthesis inhibitor HET0016 induced the same relaxant response to acetylcholine as in the nonsupplemented control group. In supplemented WKYs, atherogenic index was decreased (0.8-fold), while iNOS and COX-2-derived PGI_2_ increased acetylcholine-induced vasodilation. These effects of ground RBS may constitute a potential mechanism for preventing cardiovascular diseases.

## 1. Introduction

Vascular inflammation and increased vascular resistance play a primary role in the pathogenesis and progression of hypertension [1]. Vascular dysfunction associated with hypertension is characterized by the attenuated endothelium-dependent vasodilation, which suggests reduced bioavailability of nitric oxide (NO) either in response to decreased production or increased utilization. During this process, superoxide (O_2_^•–^), hydrogen peroxide (H_2_O_2_), or peroxynitrite (ONOO^−^) are formed. Oxidation of membrane phospholipids by these reactive species results in the production of lipid hydroperoxide molecules and isoprostanes. This in turn impairs membrane functioning, which inactivates receptors and enzymes bound with this membrane, and increases membrane permeability.

Endothelial cells are able to control the tension of arteries through the release of several vasoactive factors, which balance each other out to obtain the physiological response. Among these factors vasodilators e.g., NO, H_2_O_2_, endothelium-derived hyperpolarizing factor (EDHF), prostacyclin as well as vasoconstrictors e.g., 20-hydroxyeicosatetraenoic acid (20-HETE), isoprostanes, and prostaglandins are the most important regulators of vascular tension in arteries [2,3].

The synthesis and/or release of NO and prostaglandins share a number of similarities. The increased production of prostaglandins is partly driven by NO, suggesting that cyclooxygenase (COX) enzymes would also be the factors involved in vascular tone regulation.

The vascular dysfunction largely depends on the enzymatic antioxidant status and COX/NOS (nitric oxide synthase) activity, but this can be modulated by the dietary addition of biologically active compounds, e.g., polyphenols and polyunsaturated fatty acids (PUFAs) sourced from functional foods, e.g., berries [4,5,6], garlic [7], resveratrol [8,9], dandelion [10], fish oil [11], and many more. Raspberries are popular fruits consumed in a fresh form as well as in the form of juice or red wine. Raspberries as whole fruits contain substantial amounts of polyphenols (ellagitannins, anthocyanins, ellagic acid, and flavanols) [12] with proven cardiovascular [13,14] and anti-cancer (especially ellagitannins) activities [15,16,17]. During the processing of raspberries, anthocyanins remain in the juice, while a significant amount of biologically active compounds stay in the pomace and are discarded. At least 80% of this pomace consists of seeds, which pass intact through the gastro-intestinal tract reducing its value [18], hence the constituents of seeds (ellagitannins and fiber) are biologically unavailable until they are released by the rupture of the seed coat [5]. Raspberry seeds (RBS) consist of approximately 23% of oil enriched with PUFAs. The main ingredients of raspberry oil are essential fatty acids (C18:2 *n*-6 linoleic and C18:3 *n*-3 α-linolenic acid) with a 1.8-fold prevalence of linoleic acid [19,20,21]. These are the fatty acids that humans and other animals must obtain together with food as the organism cannot synthesize them; however, they are required for a good health.

This study points to the possible health potential of RBS through the implementation of the usually discarded seeds into a daily diet. Moreover, we propose a fine grinding method that damages the seed coat and increases the bioavailability of compounds hidden inside the seed. We aimed to assess the long-term effects of supplementation on the participation of NO and COX-derived factors in endothelium-intact vasodilation induced by acetylcholine (ACh) under normotensive and hypertensive conditions. In addition, the enzymatic antioxidant status and lipid profile of blood plasma were studied in normotensive Wistar–Kyoto rats (WKYs) and in spontaneously hypertensive rats (SHRs).

## 2. Materials and Methods

### 2.1. Plant Material

The raspberry seeds (RBS) were obtained after industrial juice production (Agro-Bio-Produkt Sp. z o.o., Grodkowice, Poland) and were dried in an SB-1.5 rotary drum dryer for biomass residues. The seeds of dried raspberries were ground in a cryogenic environment (Freezer Mill 6870 SPEX SamplePrep. Inc., Stanmore, UK) to obtain particles smaller than 0.65 mm. This process made it possible to get through the seed coat and to preserve the bioactive components (ellagitannins and lipids) hidden inside the seeds.

The AOAC (2007) methodology was used to determine the selected parameters of the basic composition of the preparations [22].

Polyphenol extraction was carried out in three steps. Extractions with acetone/water/formic acid (70/29.9/0.1, *v*/*v*) were performed. First, 500 mg of ground material was vortexed with 4 mL of solvent and sonicated (15 min). It was then centrifuged at 4800× *g*, and the extract was poured into a flask. The vortexing was repeated with 3 mL of solvent, twice.

The analysis was performed using a Smartline chromatograph (Knauer, Berlin, Germany), with a degasser (Manager 5000), two pumps (P1000), an autosampler (3950), a thermostat, and a PDA detector (2800). Ellagitannins were separated on a 250 mm × 4.6 mm i.d., 5 μm, Gemini C18 110A column (Phenomenex, Torrance, CA, USA) by gradient elution with solvent A (0.05% (*v*/*v*) phosphoric acid in water) and solvent B (63:20:17 (*v*/*v*/*v*) acetonitrile–methanol–water with 0.05% phosphoric acid). The column temperature was set to 35 °C, the flow rate was 1.25 mL/min, and the gradient program was as follows: 0–5 min, 5% (*v*/*v*) B; 5–30 min, 5–28% (*v*/*v*) B; 30–40 min, 28–73% (*v*/*v*) B; 40–45 min, 73% (*v*/*v*) B; 45–47 min, 73–5% (*v*/*v*) B; 47–56 min, 5% (*v*/*v*) B. The injection volume was 20 μL. Data were collected using ClarityChrom v. 3.0.5.505 software (Knauer, Berlin, Germany). The standards applied were ellagic acid (Extrasynthese, Genay, France), sanguiin H-6, lambertianin C, and bis-HHDP-glucose. Sanguiin H-6 (to calculate sanguiin H6 and H10 content), lambertianin C (to calculate lambertianin C and D content) and bis-HHDP-glucose were obtained as described previously [23,24].

Procyanidins were determined by the excess phloroglucinol degradation method. To a 20 mg sample was added methanol solution (0.8 mL) with phloroglucinol (75 g/L) and ascorbic acid (15 g/L). The addition of hydrochloric acid in methanol (0.4 mL of 0.2 mol/L) started the reaction, which proceeded at 50 °C for 30 min. It was stopped by the addition of sodium acetate solution (0.6 mL of a 40 mmol/L) in an ice bath. After centrifugation at 3600× *g* for 5 min and dilution with a sodium acetate solution (40 mmol/L), the samples were analyzed using a Smartline chromatograph with a P2800 UV−VIS detector (both from Knauer, Berlin, Germany), an RF-10AXL fluorescence detector (FD) (Shimadzu, Tokyo, Japan), and a Gemini 110A 5 μm C18 column (250 × 4.60 mm) (Phenomenex, Torrance, CA, USA). Phase A consisted of acetic acid and water (2.5/97.5, *v*/*v*) and phase B of acetonitrile and water (80/20, *v*/*v*). The applied gradient, with the flow rate 1 mL/min at 25 °C, was as follows: 0–10 min, 4–7% B; 10–27 min, 7–30% B; 27–29 min, 30–70% B; 29–34 min, 70% B; 34–35 min, 70–4% B; and 35–40 min, 4% B. The retention times and UV−VIS spectra were compared with those of standards (−)- epicatechin, (+)-catechin, (−)-epicatechin−phloroglucinol adduct, and (+)-catechin−phloroglucinol adduct was used for identification. The excitation wavelength was 278 nm, and the emission wavelength was 360 nm.

### 2.2. Drugs and Chemicals

The drugs used were acetylcholine (ACh) chloride, noradrenaline (NA) hydrochloride, 1400W, indomethacin, NS-398 (Sigma-Aldrich, Schnelldorf, Germany), SQ-29548, furegrelate, HET0016 (Cayman Chemical, Ann Arbor, MI, USA), and tranylcypromine (USP, Twinbrook, Rockville, Italy).

According to their solubility, the chemicals were dissolved as follows: 1400W in methanol; HET0016, SQ-29548, and indomethacin in ethanol; and NS-398 in DMSO. NA was dissolved in a mixture of sodium chloride + ascorbic acid (0.9% and 0.01% w/v). Other drugs were prepared in distilled water.

The stock solutions (10 mM) were maintained at −20 °C, and appropriate dilutions were made in a Krebs–Henseleit buffer (KH buffer: mM; NaCl 115, CaCl_2_ 2.5, KCl 4.6, KH_2_PO_4_ 1.2, MgSO_4_ 1.2, NaHCO_3_ 25, and glucose 11.1 at pH 7.4) on the day of the experiment. The maximal solvent concentration in organ baths was less than 0.01% (*v*/*v*).

### 2.3. Ethical Statements

The study was approved by the Ethics Committee for Animal Experimentation (permission no. 09/2018; Olsztyn, Poland) and was performed in accordance with the European Union Directive 2010/63/EU for animal experiments and conformed to the Guide for the Care and Use of Laboratory Animals (US National Institutes of Health Publications No. 86–26, revised 2014). All efforts were made to minimalize animal suffering. The replacement, reduction, and refinement (3Rs) rule was respected in the study.

### 2.4. Animal Protocol and Dietary Treatment

Male normotensive Wistar-Kyoto rats (WKYs/NCrl, *n* = 12) and spontaneously hypertensive rats (SHRs/NCrl, *n* = 12) from Charles River Laboratories (Sulzfeld, Germany) at 10 weeks of age were randomly divided into two groups of six animals each. Four experimental groups were studied. The control groups for WKYs and SHRs were not supplemented with RBS. The other two groups were fed with a diet supplemented with RBS (7 g/100 g of a diet) for a period of 6 weeks.

Rats were kept individually in stainless steel cages under the following conditions: temperature of 21–22 °C, a relative humidity of 50% ± 10% and a ventilation rate of 20 air changes during one hour. A fresh diet was served every day ad libitum and access to water was continuous. The experimental diets were modifications of a casein diet recommended by the American Institute of Nutrition for laboratory rodents, see Table S1.

### 2.5. Experimental Procedures

Rats were anesthetized by intraperitoneal injection of ketamine (100 mg/kg of body weight (BW)) and xylazine (10 mg/kg BW) and killed by decapitation. Blood samples were kept in tubes containing heparin + EDTA as an anticoagulant and centrifuged at 3000× *g* for 10 min to separate the blood plasma. The organs such as heart, liver, and kidneys were carefully isolated and weighed. All samples were immediately placed in liquid nitrogen (−196 °C) for 30 min and then stored at low temperature (−80 °C) for further analyses.

### 2.6. Analysis of Blood Plasma

The blood plasma lipid profile (triglyceride, total cholesterol, high-density lipoprotein cholesterol), uric acid, urea, and the activity of liver enzymes (aspartate aminotransferase (AST) and alanine aminotransferases (ALT)) were measured using a biochemical auto-analyzer (Pentra C200, Horiba, Kyoto, Japan). The obtained results were expressed as mmol/L and as U/L for liver enzymes (AST and ALT).

The activity of superoxide dismutase (SOD) was determined using Ransod and Ransel diagnostic kits (Randox), and catalase (CAT) was determined by the enzymatic decomposition of hydrogen peroxide. Data were expressed in U/mL.

### 2.7. Vascular Reactivity Studies

The thoracic aorta was immediately cleaned of adherent tissue, cut into 6–8 rings and suspended horizontally under a resting tension of 1 g in 5 mL tissue baths (Graz, Harvard Apparatus, Barcelona, Spain) containing KH buffer, aerated with carbogen (95% oxygen and 5% carbon dioxide mixture), maintained at 37 °C and at a pH of 7.4. Each ring was connected with a transducer and amplifier (F-30, TAM-A Hugo-Sachs Elecktronik, March, Germany) to measure the isometric force [25].

Aortic rings were washed 3 times in KH buffer over 60 min. Next, the aortic rings were incubated for 30 min with either iNOS inhibitor (1400W, 1 µM), nonselective COX inhibitor (indomethacin, 10 µM), selective COX-2 inhibitor (NS-398, 10 µM), PGI_2_ synthesis inhibitor (tranylcypromine, TCP, 10 µM), thromboxane receptor (TP) antagonist (SQ-29548, 1 µM), TxA_2_ synthesis inhibitor (furegrelate, 1 µM), or an inhibitor of 20-HETE formation (HET0016, 0.1 µM). The cumulative concentration response curves to ACh (0.1 nM–10 µM) were built to assess the endothelium-intact vasodilation of aortic rings precontracted with NA (1.5 ± 0.18 g). Only one cumulative concentration–response curve (CCRC) was performed on each aortic ring.

### 2.8. Data Analysis and Statistics

A nontraditional lipid profile was calculated using triglycerides (TG), total cholesterol (TC), high density lipoprotein (HDL) as follows: non-HDL = TC − HDL; TC/HDL; non-HDL/HDL, and the atherogenic index of plasma (AIP) = log_10_(TG/HDL). Carbohydrates were calculated as total solid fractions − (proteins + fat + ash).

The calculations and graphs were done and analyzed in GraphPad Prism 8.4. Endothelium-intact vasodilation to ACh was expressed as a percentage of the response to NA. The individual CCRCs were analyzed by a nonlinear regression model, which determined the area under the curve (AUC), the maximal response (E_max_, %), and the potency (the negative logarithm of the concentration causing a half-maximum effect, pEC_50_).

The Gaussian distribution of residuals and homoscedasticity of variance were tested for all data. The group comparison was performed by two-way ANOVA with post hoc test. Due to limitations on sample volume collected from animal subjects and/or data outlier detection by the Grubbs’ test, in a few cases *n* varied among bioassays. Differences were considered significant when *p* ≤ 0.05.

## 3. Results

### 3.1. Plant Material

The main component of ground RBS was dietary fiber (64%), and the main polyphenols were ellagitannins (1.2%) and flavan-3-ols (0.45%). Raspberry ellagitannins were mainly constituted by a sanguiin-H-6 (40%) and a lambertianin C (31%), see Table 1.

### 3.2. Animal Characteristics

**Figure 1:**
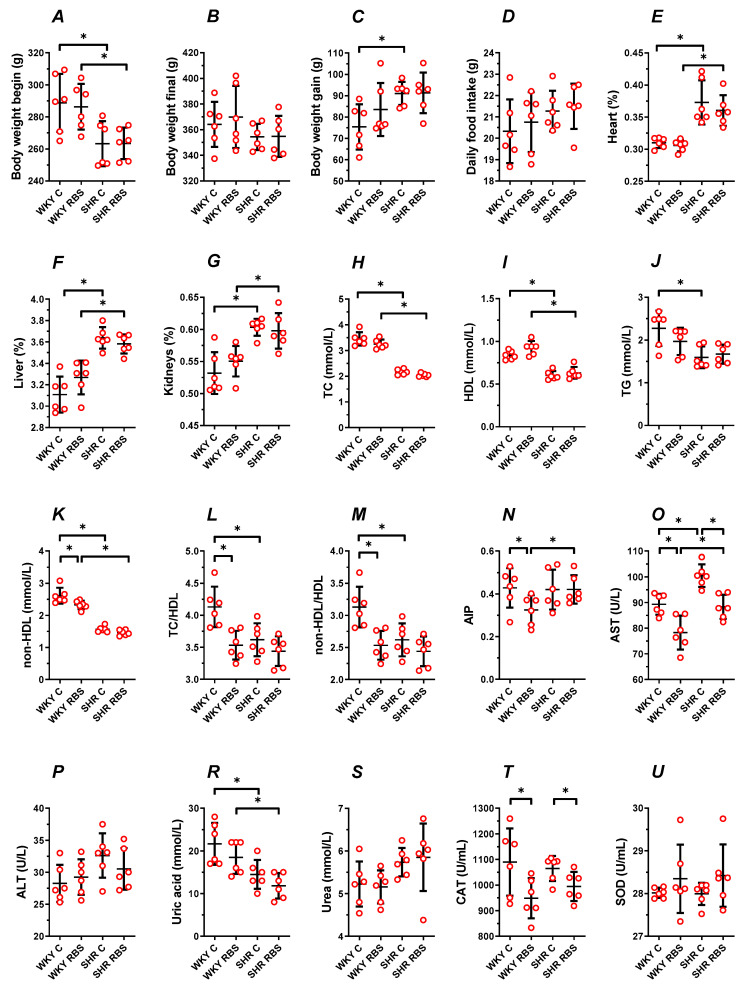
The influence of experimental diets on rat body weight (**A**–**C**), daily feed intake (**D**), weight of internal organs (**E**–**G**), the traditional lipid profile (**H**–**J**), the nontraditional lipid profile (**K**–**N**), liver enzymes (**O**,**P**), uric acid (**R**), urea (**S**), and antioxidant status (**T**,**U**). From 10 weeks of age, Wistar–Kyoto rats (WKYs) and spontaneously hypertensive rats (SHRs) were fed for 6 weeks with a diet enriched with 7% ground raspberry seeds (RBS). Values are expressed as mean ± SD, *n* = 6, * *p* ≤ 0.05 (ANOVA/Tukey’s).

Dietary supplementation with ground RBS (7 g/100 g of a diet), which delivered daily 1.47 ± 0.02 g of ground RBS, neither modified the body weight gain (*p* ≥ 0.3302, Figure 1A–C) nor changed the daily feed intake (*p* ≥ 0.8872, Figure 1D) during 6 weeks of the experiment. However, supplementation with RBS made insignificant (*p* = 0.3696) the 1.2-fold increase in the body weight gain of SHRs (*p* = 0.0192, WKYs vs. SHRs, Figure 1C). Data are summarized in Table S2.

There was no significant change in the heart, liver, and kidneys mass-to-body weight ratio of rats not supplemented and supplemented with ground RBS (*p* ≥ 0.1669, WKYs vs. WKYs + RBS and *p* ≥ 0.4728, SHRs vs. SHRs + RBS, Figure 1E–G). The observed increase in the mass of internal organs in SHRs between nonsupplemented controls (*p* ≤ 0.0058, WKYs vs. SHRs) was also observed in the RBS-supplemented groups (*p* ≤ 0.0402, WKYs + RBS vs. SHRs + RBS, Figure 1E–G); see also Table S2.

### 3.3. Blood Plasma Lipid Profile

The tested diets supplemented with ground RBS did not modify in a significant way the TC (*p* ≥ 0.1838, Figure 1H), HDL-cholesterol (*p* ≥ 0.0733, Figure 1I), and TG (*p* ≥ 0.3447, Figure 1J), compared to the respective nonsupplemented control group.

The observed decrease in TC (×0.63-fold SHRs, Figure 1H) and HDL-cholesterol (×0.72-fold SHRs, Figure 1I) between nonsupplemented controls (*p* ≤ 0.0058) were also observed in RBS-supplemented rats (×0.63- and ×0.68-fold, respectively), see Table S3. However, RBS supplementation made insignificant (*p* = 0.3628) the 0.7-fold decrease of TG in SHRs (*p* = 0.0078, WKYs vs. SHRs, Figure 1J).

RBS supplementation decreased the calculated atypical lipid profile in WKYs. This was observed as decreased non-HDL-cholesterol (×0.9-fold, *p* = 0.0173, Figure 1K), both the TC/HDL and non-HDL/HDL (×0.8-fold, *p* = 0.0036, Figure 1L,M) and AIP (×0.76-fold, *p* = 0.05, Figure 1N). Such changes were not observed in supplemented SHRs (*p* ≥ 0.0625).

In control rats, supplementation with RBS made insignificant (*p* ≥ 0.4891) the observed decrease in TC/HDL and non-HDL/HDL (×0.8-fold, *p* = 0.0117, WKYs vs. SHRs), see Table S3. Meanwhile, AIP increased in supplemented SHRs (×1.3-fold, *p* = 0.0342, WKYs + RBS vs. SHRs + RBS).

### 3.4. AST, ALT, Uric Acid, and Urea

Dietary supplementation with RBS markedly decreased by 0.88-fold the blood plasma AST in normotensive and hypertensive rats (*p* = 0.0095, WKYs and *p* = 0.0045, SHRs, Figure 1O). A significant decrease of AST was also observed in studied WKYs compared to SHRs (×0.89-fold, *p* ≤ 0.0183) regardless of the RBS intake, see Table S4.

Dietary RBS neither modified the ALT (*p* ≥ 0.5888, Figure 1P) nor the uric acid (*p* ≥ 0.2776, Figure 1R) and urea (*p* ≥ 0.9873, Figure 1S) in studied groups. In SHRs, the observed decrease of uric acid between control groups (×0.67-fold, *p* = 0.003, WKYs vs. SHRs, Figure 1R) remained decreased after RBS supplementation (×0.64-fold, *p* = 0.0062, WKYs + RBS vs. SHRs + RBS), see Table S4.

### 3.5. The Enzymatic Antioxidant Status

Supplementation with RBS markedly decreased the activity of CAT in normotensive and hypertensive rats (×0.87-fold WKYs, *p* = 0.0468 and ×0.93-fold SHRs, *p* = 0.0390, Figure 1T), whereas superoxide dismutase (SOD) remained unmodified (*p* ≥ 0.1797, Figure 1U), see Table S4.

### 3.6. Vascular Reactivity Studies

**Figure 2:**
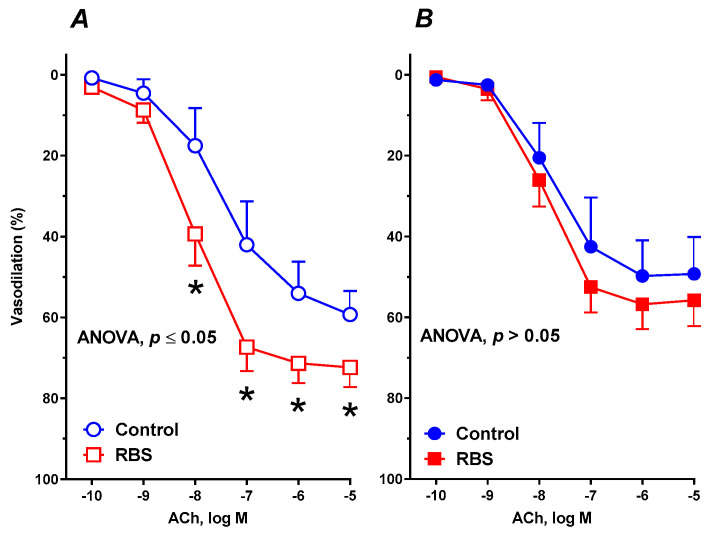
The influence of experimental diets on the vasodilator response to acetylcholine (ACh) in thoracic arteries isolated from WKYs (**A**) and SHRs (**B**). From 10 weeks of age, rats were fed a control diet and with a diet supplemented for 6 weeks with added 7% ground RBS. Results (mean ± SEM) are expressed as a percentage of the inhibition of the contraction induced by noradrenaline (1.5 ± 0.18 g), * *p* ≤ 0.05 (two-way ANOVA/Sidak’s), m = 6–8.

After 6 weeks of supplementation with ground RBS, vasodilation induced by ACh was enhanced in WKYs (Figure 2A). In contrast, in SHRs, vasodilation to ACh was comparable to that observed in untreated rats (Figure 2B). A significant difference was also observed between supplemented WKYs and SHRs, which was not observed between the controls (Table 2).

**Figure 3:**
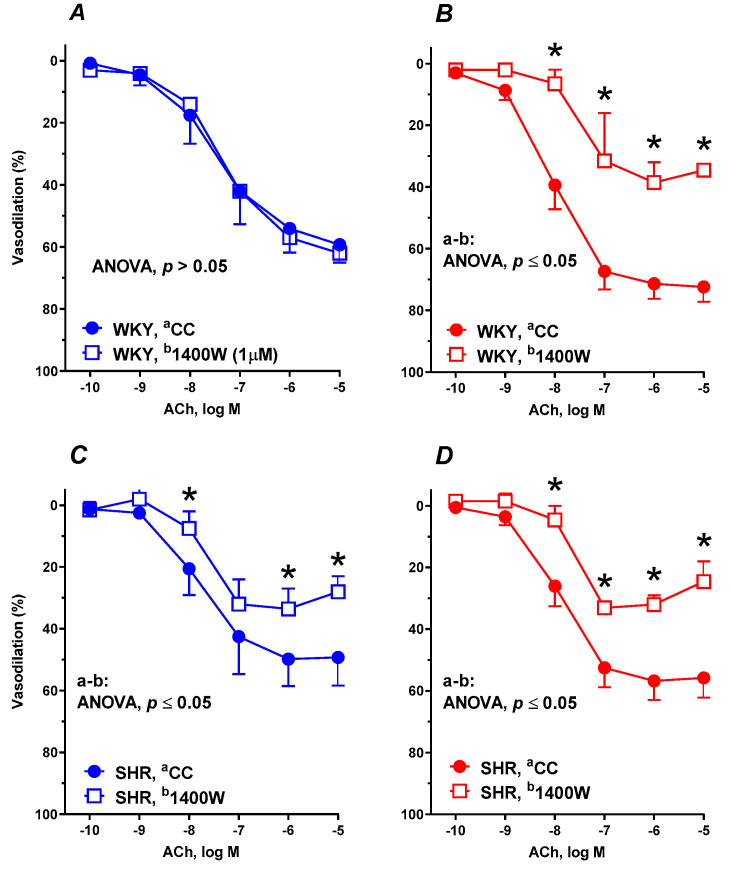
The influence of experimental diets on the vasodilator response to acetylcholine in thoracic arteries isolated from WKYs (**A**,**B**) and SHRs (**C**,**D**). From 10 weeks of age, rats were fed a control diet without added raspberry seeds (RBS) (**A**,**C**) and with a diet supplemented for 6 weeks with added 7% ground RBS (**B**,**D**). ACh-induced vasodilation was analyzed in the absence and presence of the selective inducible nitric oxide synthase (iNOS) inhibitor (1400W, 1 μM, 30 min). Results (mean ± SEM) are expressed as a percentage of the inhibition of the contraction induced by noradrenaline (1.5 ± 0.18 g), * *p* ≤ 0.05 (two-way ANOVA/Tukey’s), *m* = 6–8.

Pre-incubation with the selective iNOS inhibitor 1400W (1 μM) did not modify the vasodilator response to ACh in control WKYs (Figure 3A), meanwhile supplementation with RBS diminished the vascular response to ACh (Figure 3B).

In contrast, in arteries from SHRs, 1400W attenuated the vasodilator response to ACh in aortic rings from both control rats (Figure 3C) and RBS-supplemented rats (Figure 3D). For further details see Table 2.

**Figure 4:**
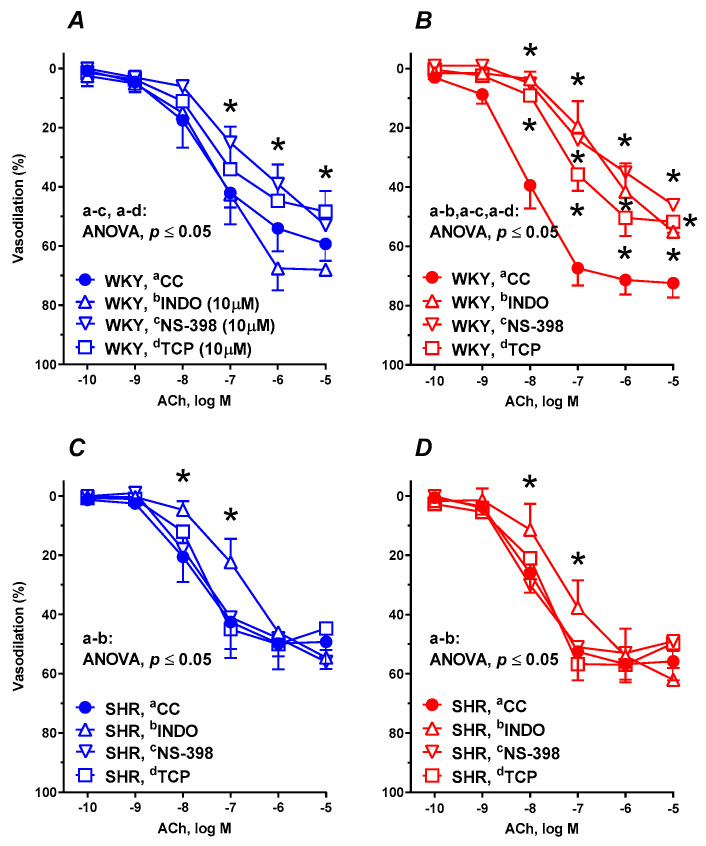
The influence of experimental diets on the vasodilator response to acetylcholine in thoracic arteries isolated from WKYs (**A**,**B**) and SHRs (**C**,**D**). From 10 weeks of age, rats were fed a control diet without added raspberry seeds (RBS) (**A**,**C**) and with a diet supplemented for 6 weeks with added 7% ground RBS (**B**,**D**). ACh-induced vasodilation was analyzed in the absence and presence of nonselective cyclooxygenase (COX) inhibitor (indomethacin, 10 µM, 30 min), selective COX-2 inhibitor (NS-398, 10 µM, 30 min), and prostacyclin (PGI_2_) synthesis inhibitor (tranylcypromine) TCP, 10 µM, 30 min). Results (mean ± SEM) are expressed as a percentage of the inhibition of the contraction induced by noradrenaline (1.5 ± 0.18 g), * *p* ≤ 0.05 (two-way ANOVA/Tukey’s), *m* = 6–8.

Pre-incubation of aortic rings from control WKYs with either COX-2 inhibitor NS-398 (10 µM) or the specific PGI_2_ synthesis inhibitor TCP (10 µM) diminished the ACh-induced vasodilation. In contrast, the nonselective COX inhibitor, indomethacin (10 µM), did not modify the vasodilation (Figure 4A). Supplementation with RBS decreased the maximal vasodilation to indomethacin (×0.75) as well as to NS-398 (×0.59-fold) and to TCP (×0.72-fold) (Figure 4B and Table 2).

In SHRs, pre-incubation with indomethacin decreased the vasodilator response to ACh in both nonsupplemented (Figure 4C) and supplemented (Figure 4D) groups of rats in a comparable way, meanwhile the response to NS-398 and TCP was not modified (see Table 2).

**Figure 5:**
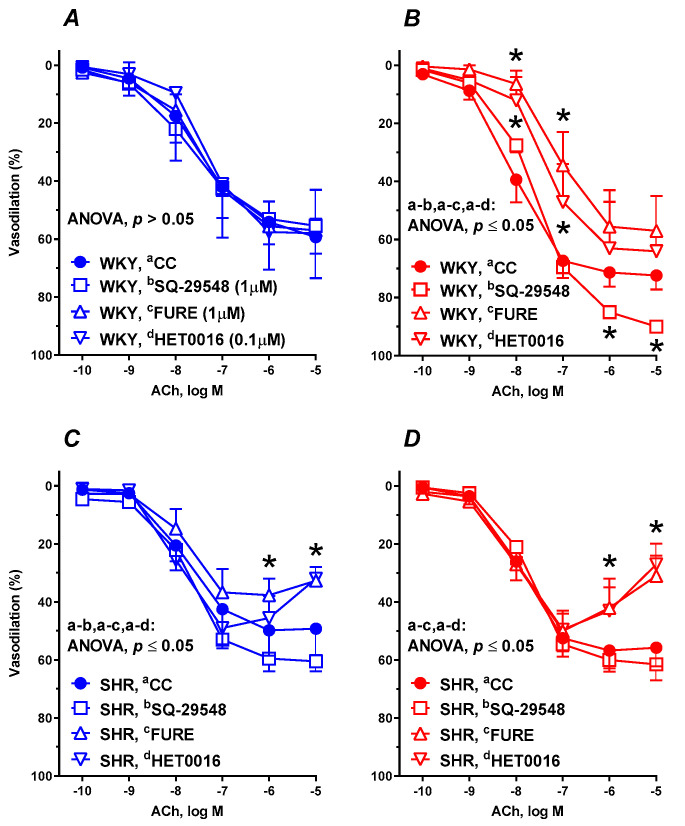
The influence of experimental diets on the vasodilator response to acetylcholine in thoracic arteries isolated from WKYs (**A**,**B**) and SHRs (**C**,**D**). From 10 weeks of age, rats were fed a control diet without added raspberry seeds (RBS) (**A**,**C**) and with a diet supplemented for 6 weeks with added 7% ground RBS (**B**,**D**). ACh-induced vasodilation was analyzed in the absence and presence of the thromboxane receptor (TP) antagonist (SQ-29548, 1 µM, 30 min), TxA_2_ synthesis inhibitor (furegrelate, 1 µM, 30 min) and an inhibitor of 20-HETE formation (HET0016, 0.1 µM, 30 min). Results (mean ± SEM) are expressed as a percentage of the inhibition of the contraction induced by noradrenaline (1.5 ± 0.18 g), * *p* ≤ 0.05 (two-way ANOVA/Tukey’s), *m* = 6–8.

Pre-incubation of aortic rings with the TP receptor antagonist (SQ-29548, 1 µM, 30 min), TxA_2_ synthesis inhibitor (furegrelate, 1 µM, 30 min), and an inhibitor of 20-HETE formation (HET0016, 0.1 µM, 30 min) did not modify the vasodilation in the control group of WKYs (Figure 5A). In RBS-supplemented WKYs, SQ-29548 enhanced, while furegrelate and HET0016 decreased, the ACh induced vasodilation (Figure 5B).

In both nonsupplemented (Figure 5C) and supplemented (Figure 5D) SHRs, pre-incubation with SQ-29548 did not modify the vascular response, meanwhile furegrelate and HET0016 decreased the maximal vasodilation.

For the AUC, E_max_, and pEC_50_ see Table 2.

## 4. Discussion

The fine grinding of raspberry seeds (RBS) applied in this study released the accumulated compounds such as ellagitannins and lipid fractions (linoleic, α-linolenic acids) from the seeds, hence this increased the level of antioxidants and available fat composed of n-3 and n-6 PUFAs (see Introduction). The seeds used in the experimentation were abundant in ellagitannins (1.2%) and flavan-3-ols (0.45%). Raspberry ellagitannins, a class of hydrolysable tannins, mainly consisted of sanguiin H-6 (40%) and lambertianin C (31%). A similar composition of RBS was obtained by Kosmala et al. [5]. In a previous study, we have pointed to the beneficial role of ground seeds within raspberry pomace compared to seedless pomace, which beneficially had modulated metabolic disorders induced by a high-fat diet [18,26]. In this study, we further analyzed the supplementation with ground RBS (1.47 ± 0.02 g per day).

Six weeks of dietary supplementation neither modified the body weight gain nor changed the daily feed intake of experimental rats. However, supplementation with ground RBS made insignificant the observed difference in the body weight gain between normotensive and hypertensive controls. There was also no significant change in the heart, liver, and kidneys mass-to-body weight ratio of rats not supplemented and supplemented with ground RBS. The observed increase in the mass of internal organs in hypertensive controls was also observed in the supplemented hypertensive rats. The above results are in accordance with the observations carried out on copper-deficient Wistar rats supplemented with another polyphenolic compound, resveratrol [8].

In our study, the typical lipid profile measured as TC, TG, and HDL-cholesterol was not modified during supplementation with ground RBS. However supplementation made insignificant the observed decrease of TG in hypertensive controls compared to normotensive rats. Similarly, Pieszka et al. [27] have not found any significant effects of raspberry oil on the lipid profile. However other authors have observed a TG-lowering effect with dietary raspberry oil [5,12,28]. The observed differences between studies might be due to the fact that RBS consist of approximately 23% of oil; so perhaps the daily dose of pure raspberry oil in our study was lower compared that in to the above-mentioned studies. Another possible explanation is a different duration of the experimental feeding. The nontraditional lipid profile is a novel index used as an indicator of dyslipidemia and associated disorders (e.g., cardiovascular diseases). Recently, the calculated atherogenic index has been postulated to be a better biomarker for coronary artery disease and type 2 diabetes than the conventional lipid profile [29,30]. Our findings revealed that supplemented normotensive rats had a lower risk of obesity and cardiovascular complications compared to nonsupplemented subjects, as indicated by the decreased atherogenic level. Furthermore, Kosmala et al. have previously reported decreased atherogenic index in rats supplemented with ground RBS [5].

Blood plasma AST, ALT, uric acid, and urea are the indicators of properly functioning metabolism with a focus on the liver and kidneys. In our study, supplementation with ground RBS decreased the plasma AST activity in both normotensive and hypertensive rats providing evidence of beneficial hepatoprotective properties. However, the ALT activity, uric acid, and urea concentrations remained unmodified. Decreased hepatotoxicity markers (decreased AST and ALT) have been previously observed in Wistar rats supplemented with raspberry oil [19]. Moreover, in the same study this was not observed in rats supplemented with an atherogenic diet (high-fat/low-fiber diet), which strengthens the hypothesis of a preventive rather than a curative effect. Liver functioning markers (AST and ALT) are altered not only due to hepatic dysfunction but also due to increased oxidative stress [31]. In our study, hypertensive rats were characterized by increased AST compared to that of the normotensive group, which points to the development of some unwanted processes during hypertension. These results coincide well with the studies of Akinnuga et al. in which the induction of diabetes resulted in an increase of liver enzymes (AST and ALT) and lipid peroxidation [32].

In low doses, PUFAs, which are present in seeds, can exert beneficial antioxidant effects on the vascular endothelium. In contrast, high doses of PUFAs, due to the high degree of unsaturation may even trigger the inflammatory processes, especially when the liver is damaged [11]. Recently, a few studies have shown the beneficial antioxidant effects of raspberry oil. Likewise, Fotschki et al. [19] and Pieszka et al. [27] studied the antioxidant properties of raspberry oil on the cellular glutathione disulfide concentration in erythrocytes and in the liver in vivo, while Teng et al. [20,21] examined the beneficial influence of raspberry oil on cellular antioxidant enzyme activities and reactive oxygen species (ROS) formation in vitro. In another study, the constituents of RBS extract have been defined as an important factors in the prevention of oxidative lymphocyte damage by ROS and a reduced level of DNA damage [33].

In our study, the enzymatic antioxidant status was modified during supplementation with ground RBS. While superoxide dismutase (SOD) remained unmodified, the catalase (CAT) activity was decreased. SOD scavenges superoxide anions (O_2_^•–^) to hydrogen peroxide (H_2_O_2_) whereas CAT further converts harmful H_2_O_2_ into water (H_2_O) and oxygen (O_2_). Decreased activity of CAT, as was observed in our study, may point to the decreased scavenging of H_2_O_2_ that may be generated in a smaller amount. In our previous study with another polyphenolic compound, resveratrol, CAT activity was also decreased during dietary supplementation of normotensive Wistar rats [8,9].

During pathological conditions (hypertension, diabetes, aging, malnutrition, or hypercholesterolemia) endothelial dysfunction exists [34,35,36,37]. This may include reduced NO bioavailability and an increase of vasoconstrictor factors which alter NOS and COX activities and the vasodilator response of isolated arteries.

In hypertensive rats, preincubation with iNOS inhibitor 1400W, nonselective COX inhibitor, indomethacin; selective COX-2 inhibitor, NS-398; PGI_2_ synthesis inhibitor, TCP; TP receptor antagonist, SQ-29548; TxA_2_ synthesis inhibitor, furegrelate; and 20-HETE synthesis inhibitor, HET0016, induced the same vasodilator response to ACh in the nonsupplemented control group as in the supplemented group. These results indicate that the hypertension was too overwhelming to benefit from RBS intake. However, in normotensive rats the RBS intake did modify the vasodilator response to ACh.

We observed that in the aortic rings from normotensive rats, 1400W reduced the ACh-induced vasodilation, however only in supplemented rats. In hypertensive rats, the vasodilation was reduced to a similar level in both supplemented and nonsupplemented rats. In healthy states, the expression of iNOS is absent [35]. Rather it occurs under inflammatory conditions as was observed in control hypertensive rats. Increased NO production generated by iNOS was also observed in mesenteric arteries of stroke-prone hypertensive rats [1] and lipopolysaccharide (LPS)-treated rats [38]. Thus, the effect observed in supplemented normotensive rats was unexpected, and further studies, including *iNOS/eNOS* gene expression, are necessary to explain this phenomenon. Meanwhile, recent studies indicate that the absence or the inhibition of iNOS may even worsen the pathology, thus it may be that iNOS, in some cases, is protective [39]. Our results are further confirmed by the induction of *iNOS* gene expression within the arterial wall after short-term oral administration of red wine polyphenolic compounds [40].

As NO synthesis by iNOS contributes to the upregulation of COX-2 [39], we did also study the involvement of the COX pathway in ACh-induced vasodilation. The synthesis and release of NO and prostaglandins share a number of similarities. The increased production of prostaglandins is partly driven by NO, suggesting that COX enzymes would be the factors involved in NO modulation. However, the evidence that COX-2 upregulates iNOS is still under investigation.

In our study, we analyzed the possible participation of arachidonic acid derivatives in ACh-induced vasodilation of supplemented rats. This was confirmed by the attenuation of ACh-induced vasodilation in aortic rings from normotensive rats in the presence of the nonselective COX inhibitor indomethacin. Because the presence of the selective COX-2 inhibitor, NS-398, decreased the ACh response to a similar extent, the vasodilator factors are derived from COX-2. This is further confirmed by the fact that aortas from polyphenolic compounds-treated rats but not those from control rats have displayed a marked expression of the COX-2 isoform [40]. PGI_2_ is a potent vasodilator and inhibitor of platelet aggregation. In our study, the PGI_2_ synthesis inhibitor, TCP, decreased the vascular response to ACh, in a similar way to indomethacin and NS-398, pointing toward PGI_2_ as a major vasodilator. In contrast, in hypertensive rats the maximal vasodilator response to ACh in the presence of indomethacin, NS-398, and TCP was not altered, which indicates that the vasodilator net effect of prostanoids in hypertensive rats has been lost. The observed upregulation of PGI_2_ might play a crucial role in the increased vasodilator response to ACh induced by ground RBS in normotensive rats, thus, enhanced production of this vasodilator agent might add to that of NO and confer additional vasoprotective benefits. This has been previously demonstrated on isolated rat aortic rings subjected to wild-artichoke extract [41].

As indicated by the results in the presence of 1400W and TCP, the increased release of vasoconstrictor prostanoids might also be involved in the ACh-induced response to counterbalance the increased vasodilation due to the enhanced production of NO and PGI_2_. Through the activation of TP receptors PGI_2_, prostaglandins, isoprostanes, and 20-hydroxyeicosatetraenoic acid (20-HETE) participate in the endothelial dysfunction associated with cardiovascular risk factors. In our study, incubation with a TP receptor antagonist, SQ-29548, increased the vasodilation in normotensive rats supplemented with ground RBS, suggesting the participation of a potent vasoconstrictor through the TP receptor. As there is an interplay between PGI_2_ and TxA_2_, we examined the influence of TxA_2_ on vascular response. However, the present results showed that, in the aorta from normotensive rats, TxA_2_ is not an important vasoconstrictor candidate [3]. Surprisingly, the TxA_2_ synthesis inhibitor, furegrelate, decreased the response in normotensive rats. The experiments with SQ-29548 and furegrelate indicate that when TxA_2_ synthesis is inhibited the production of other vasoconstrictors, whose action is mediated by TP receptor activation, might be increased.

In addition, the participation of the vasoconstrictor 20-HETE, originating from the cytochrome P450 family, can also be suggested [3]. 20-HETE alters endothelial cell barrier integrity [42] so the CYP/20-HETE pathway is an important therapeutic target for the prevention of cardiovascular disease [43]. However, in the presence of the 20-HETE synthesis inhibitor, HET0016, we observed a decreased response in normotensive and hypertensive rats, which was similar to the result observed in the presence of the TxA_2_ synthesis inhibitor, furegrelate. Consequently, the participation of other vasoconstrictor prostanoids, which can be synthetized through COX-2 and activate the TP receptor, can be proposed. Another possible explanation is that TxA_2_ and 20-HETE may upregulate the aforementioned iNOS. Thus further studies are needed.

Furthermore, certain isoprostanes, which are formed by a direct free radical attack on arachidonic acid, are able to constrict arteries by activating TP receptors, however this was not studied during this experiment.

Dietary consumption of fruits, vegetables, or red wine is inversely associated with morbidity and mortality from coronary heart disease [44]. In vitro studies have shown that this beneficial effect may be in part explained by the presence of polyphenols. Indeed, polyphenols increase the generation of PGI_2_ and NO from vascular endothelium and decrease platelet aggregation. These in the longer term do induce the expression of genes (e.g., *Nox-1*) protecting the cardiovascular system [40,45]. The new proposed mechanism of polyphenols focus on “para-hormesis” effect where endogenous defense systems are activated to provide increased protection against subsequent stress challenges [46]. Our study provides further justification to the advice to consume wild fruits and vegetables [41] and suggests that close attention should be paid to the diet and its bioactive components, including ellagitannins because it can effectively modulate important parameters that are in control of cardiovascular system. Further studies should be focused on duration of supplementation and on a dose–response phenomenon characterized by a low-dose stimulation, high-dose inhibition.

## 5. Conclusions

Hypertensive control rats showed significant changes in blood plasma and in the reactivity of thoracic arteries as manifested by an increased AST and decreased TG, TC, and HDL-cholesterol. A decreased vascular response to ACh with increased participation of the vasoconstrictor mechanism was also observed. From the above-mentioned discrepancies, only AST was improved in supplemented hypertensive rats. In normotensive rats, supplementation with ground RBS additionally modulated vascular functioning and increased vasodilation in response to ACh. Supplementation with ground RBS increased the participation of NO derived from iNOS in ACh-induced vasodilation and also increased participation of PGI_2_ derived from COX-2 in the vasodilator response. However, the compensatory effects of other vasoconstrictors in the vascular tone regulation through TP receptors is highly indicated and that requires further studies.

## Figures and Tables

**Table 1:** The characterization of ground raspberry seeds.

Item	Value (%)
Dry matter content	96.52 ± 0.18
Ash	1.71 ± 0.06
Protein	10.51 ± 0.45
Fat	14.14 ± 0.22
Carbohydrates *	68.50
Total dietary fiber	63.92 ± 0.38
Soluble dietary fiber	1.23 ± 0.02
Insoluble fiber	62.69 ± 0.40
Total polyphenols	1.66 ± 43.6
Total polyphenols	mg/100 g
Ellagitannins	1211.3 ± 34.4
bis-HHDP-glucose isomer 1	34.6 ± 3.2
bis-HHDP-glucose isomer 2	38.9 ± 2.4
Sanguiin H-10 isomer 1	31.8 ± 0.5
Lambertianin C without ellagic acid	29.1 ± 1.4
Sanguiin H-10 isomer 2	31.1 ± 1.0
Lambertianin C isomer 1	21.5 ± 0.7
Lambertianin C isomer 2	41.9 ± 0.0
Lambertianin C isomer 3	14.4 ± 0.8
Lambertianin D	113.5 ± 3.0
Lambertianin C	375.8 ± 7.0
Sanguiin H-6	478.8 ± 14.5
Ellagic acid	106.9 ± 4.8
Flavan-3-ols, including	453.95 ± 9.22
Procyanidins	439.03 ± 8.76
Free catechins	14.92 ± 0.46

* Carbohydrates = dry matter − (ash + protein + fat + total polyphenols). Soluble dietary fiber content was calculated as total dietary fiber minus insoluble dietary fiber. Values are expressed as the mean ± SD, *n* = 3.

**Table 2:** Changes in the area under the curve (AUC), maximal responses (E_max_), and pEC_50_ (potency) of an intact thoracic aorta from rats fed with experimental diets for 6 weeks in response to acetylcholine.

	Experimental Group															
	WKYs, Control				WKYs, RBS				SHRs, Control				SHRs, RBS			
	m	AUC	E_max_ (%)	pEC_50_	m	AUC	E_max_ (%)	pEC_50_	m	AUC	E_max_ (%)	pEC_50_	m	AUC	E_max_ (%)	pEC_50_
ACh *	8	148.0 ± 24.12	57.08 ± 4.917	7.502 ± 0.2742	8	224.3 ± 14.66 #	72.43 ± 2.908 #	8.055 ± 0.1353 #	8	140.5 ± 26.21	49.66 ± 4.965	7.823 ± 0.3193	8	166.9 ± 17.33 #€	57.1 ± 3.235 €	7.942 ± 0.1798
+1400W	6	149.5 ± 21.3	60.95 ± 0.9386	7.322 ± 0.04617	6	96.75 ± 17.46 *#	37.77 ± 4.492 *#	7.50 ± 0.3692 *	6	87.31 ± 12.19 *	32.64 ± 3.688 *	7.717 ± 0.3405	6	82.88 ± 7.028 *	30.93 ± 3.191 *	7.739 ± 0.295
+Indo	6	165.3 ± 9.624	70.09 ± 3.564	7.184 ± 0.1264	6	94.25 ± 13.02 *#	54.38 ± 4.446 *#	6.633 ± 0.1993 *#	6	100.8 ± 10.57 *	54.3 ± 3.673	6.827 ± 0.1419 *	6	136.2 ± 21.52 *#	59.97 ± 5.512	7.215 ± 0.255 *
+NS-398	6	99.50 ± 18.98 *	49.57 ± 3.583	6.913 ± 0.1835 *	6	87.67 ± 16.44 *	42.72 ± 2.794 *	7.091 ± 0.1647 *	6	135.1 ± 11.34	52.07 ± 2.476	7.687 ± 0.1463	6	162.5 ± 21.11 #	52.18 ± 1.568	8.159 ± 0.09929
+TCP	6	118.2 ± 25.26 *	47.54 ± 5.896 *	7.377 ± 0.3778	6	123.3 ± 10.88 *	52.33 ± 2.62 *	7.316 ± 0.1437 *	6	130.7 ± 10.98	49.26 ± 2.667	7.675 ± 0.1672	6	166.2 ± 13.10 #	56.21 ± 3.457	7.855 ± 0.2007
+SQ-29548	6	177.9 ± 37.19	63.03 ± 7.603	7.641 ± 0.396	6	233.8 ± 32.669 *#	88.34 ± 0.9784 *#	7.592 ± 0.03549 *	6	172.5 ± 7.331	61.09 ± 1.841	7.704 ± 0.1008	6	169.0 ± 8.703	62.01 ± 2.238	7.749 ± 0.1134
+FURE	6	149.8 ± 16.27	57.18 ± 4.771	7.457 ± 0.2676	6	126.2 ± 21.36 *	58.39 ± 6.11 *	7.147 ± 0.2344 *	6	109.3 ± 14.76 *	36.86 ± 3.14 *	7.867 ± 0.2796	6	141.2 ± 17.23 *#	41.21 ± 4.321 *	8.319 ± 0.3902 *#
+HET0016	6	140.3 ± 7.080	59.31 ± 2.119	7.322 ± 0.1028	6	159.5 ± 26.36 *	65.13 ± 7.552 *	7.367 ± 0.3457 *	6	138.0 ± 10.64	42.66 ± 3.8	8.231 ± 0.2997 *	6	137.0 ± 11.57 *	40.07 ± 4.368 *	8.331 ± 0.3965 *

Data are expressed as mean ± SEM of *n* = 6 rats and *m* replicates analyzed by a nonlinear regression model. * vs. control conditions (ACh). # vs. nonsupplemented control. € vs. respective WKYs (p ≤ 0.05, two-way ANOVA/Tukey’s). ACh—acetylcholine; AUC—area under the curve; E_max_—maximal response values expressed as a percentage of dilation; Indo—indomethacin; L-NAME—N(ω)-nitro-L-arginine methyl ester; pEC_50_—(-logEC_50_) drug concentration exhibiting 50% of the E_max_ expressed as negative log molar; SNP—sodium nitroprusside.

## Supplementary Materials

The following are available online at https://www.mdpi.com/2072-6643/12/6/1630/s1, Table S1: Composition of experimental diets fed to rats; Table S2: Daily dietary intake, body weight change, and organ weights of experimental rats; Table S3: Traditional and nontraditional lipid profile of experimental rats; Table S4: Blood plasma biochemical indices.
