# Supplementary material for: The Characterization of Ground Raspberry Seeds and the Physiological Response to Supplementation in Hypertensive and Normotensive Rats
Source: Nutrients. 2020 Jun 1;12(6):1630. doi: 10.3390/nu12061630 (PMC7352221; doi:10.3390/nu12061630)
Supplement: Supplementary file 1 [file nutrients-12-01630-s001.pdf]

Table S1. Composition of experimental diets fed to rats.

Ingredient (g/kg)	Control diet	Diet with added ground RBS
Casein [a]	200	200
DL-methionine	3.0	3.0
Celullose [b]	50	6.1
Sucrose	100	100
Rapeseed oil [c]	20	10.1
Lard	60	60
Ground raspberry seeds	-	70
Mineral mixture [d]	35	35
Vitamin mixture [e]	10	10
Choline chloride	2.0	2.0
Corn starch	520	503.8
Calculated content of polyphenols	0	1.2

[a] Casein preparation (LACPOL Co., Murowana Goslina, Poland), containing (%): Crude protein (89.7), Crude fat (0.3), Ash (2.0), and water (8.0).

[b] α-cellulose (Sigma-Aldrich, St. Louis, MO, USA), main source of dietary fiber.

[c] rapeseed oil – Majewski et al. [11]

[d] AIN-93G-MX, containing (%): Calcium Carbonate anhydrous (35.7), Potassium Phosphate monobasic (19.6), Potassium Citrate (7.078), Sodium Chloride (7.4), Potassium Sulfate (4.66), Magnesium Oxide (2.4), Ferric Citrate (0.606), Zinc Carbonate (0.165), Sodium meta-silicate 9H2O (0.145), Manganous Carbonate (0.063), Cupric Carbonate (0.03), Chromium Potassium Sulfate·12H2O (0.0275), Boric Acid (17.5% B) (0.00815), Sodium Fluoride (45.24% F) (0.00635), Nickel Carbonate (0.00318), Lithium Chloride (0.00174), Sodium Selenate anhydrous (0.001025), Potassium Iodate (0.0010), Ammonium Paramolybdate·4H2O (0.000795), Ammonium Vanadate (0.00066), powdered Sucrose (22.1026).

[e] AIN-93G Vitamin Mix, containing (%):Nicotinic Acid (0.3), Calcium Pantothenate (0.16), Pyridoxine HCl (0.07), Thiamin HCl (0.06), Riboflavin (0.06), Folic Acid (0.02), Biotin (0.002), Vitamin B12 (Cyanocobalamin, 0.1% in mannitol) (0.25), Vitamin E (all-rac-α-tocopheryl Acetate, 500 IU/g) (1.50), Vitamin A (all-trans-retinyl Palmitate, 500,000 IU/g) (0.08), Vitamin D3 (Cholecalciferol, 400,000 IU/g) (0.025), Vitamin K1 (Phylloquinone) (0.0075), powdered Sucrose (97.4655).

Table S2. Daily dietary intake, body weight change and organ weights of experimental rats.

Experimental group	Food intake/day (g) [a]	RBS intake/day (g) [b]	Initial body weight (g)	Final body weight (g)	Body weight gain (g) [c]	Organs weight (g/100 g BW) [d]		
						Heart	Liver	Kidneys
WKY, Control<br>(C)	20.33 ± 0.608<br>19.91<br>(19.26–21.60)	1.423 ± 0.043<br>1.394<br>(1.348–1.512)	288.8 ± 7.403<br>290.3<br>(269.4–307.5)	364.1 ± 7.16<br>366.5<br>(349.5–376.3)	75.35 ± 4.35<br>76.70<br>(65.13–84.15)	0.306 ± 0.004<br>0.306<br>(0.299–0.314)	3.11 ± 0.07<br>3.09<br>(2.96–3.23)	0.532 ± 0.013<br>0.517<br>(0.508–0.563)
WKY, Raspberry Seeds<br>(RBS)	20.76 ± 0.572<br>21.33<br>(19.14–21.78)	1.453 ± 0.040<br>1.493<br>(1.340–1.525)	286.3 ± 5.811<br>287.1<br>(273.1–298.7)	369.9 ± 9.89<br>362.8<br>(349.7–397.8)	83.52 ± 5.07<br>76.70<br>(75.53–95.30)	0.306 ± 0.004<br>0.308<br>(0.297–0.312)	3.27 ± 0.06<br>3.31<br>(3.15–3.40)	0.551 ± 0.010<br>0.556<br>(0.536–0.563)
SHR, Control<br>(C)	21.27 ± 0.388<br>20.98<br>(20.55–22.06)	1.489 ± 0.027<br>1.469<br>(1.438–1.544)	263.3 ± 5.749<br>262.0<br>(250.6–276.1)	354.4 ± 4.187<br>353.4<br>(344.4–365.3)	91.05 ± 2.22<br>92.75<br>(84.85–94.55)	0.373 ± 0.014<br>0.356<br>(0.348–0.414)	3.64 ± 0.04<br>3.62<br>(3.58–3.71)	0.603 ± 0.005<br>0.607<br>(0.596–0.612)
SHR, Raspberry Seeds<br>(RBS)	21.50 ± 0.432<br>21.54<br>(20.97–22.42)	1.505 ± 0.030<br>1.508<br>(1.468–1.570)	263.4 ± 4.012<br>264.8<br>(252.1–273.0)	354.7 ± 6.535<br>352.4<br>(340.3–369.5)	91.30 ± 3.89<br>92.50<br>(83.43–97.58)	0.360 ± 0.010<br>0.357<br>(0.341–0.377)	3.58 ± 0.04<br>3.59<br>(3.51–3.66)	0.598 ± 0.011<br>0.592<br>(0.578–0.622)
p–value								
WKY, C vs. WKY, RBS	0.8872		0.7999	0.9398	0.3302	0.3904	0.1669	0.6446
SHR, C vs. SHR, RBS	0.9802		0.8506	>0.9999	>0.9999	0.4728	0.8643	0.9839
WKY, C vs. SHR, C	0.4171		**0.0216**	0.7663	**0.0192**	**0.0058**	**<0.0001**	**0.0020**
WKY, RBS vs. SHR, RBS	0.6099		**0.0088**	0.4585	0.3696	**0.0022**	**0.0032**	**0.0402**

[a] Daily total food intake per animal during 6 weeks of supplementation.
[b] Daily RBS intake per animal during 6 weeks of supplementation.
[c] Calculated as: final body weight (g) – initial body weight (g).
[d] The weight of the internal organs calculated as: organ weight (g)/final body weight (g)*100 (g).
Data are expressed either as means ± SEM or the median (with Q1 and Q3), of n = 6 rats: two-way ANOVA/Tukey's. Bold values indicate statistically significant differences ($p \leq 0.05$).
Abbreviations: BW, body weight; RBS, dried raspberry seeds.

Table S3. Traditional and nontraditional lipid profile of experimental rats.

	Traditional lipid profile			Nontraditional lipid profile [a]			
Experimental group	TC (mmol/L)	HDL (mmol/L)	TG (mmol/L)	non-HDL = TC – HDL (mmol/L)	TC/HDL	*non*HDL/HDL	AIP = logTG/HDL
WKY, Control<br>(C)	3.45 ± 0.11<br>3.42<br>(3.25–3.61)	0.837 ± 0.021<br>0.830<br>(0.7875–0.8875)	2.273 ± 0.168<br>2.480<br>(1.825–2.535)	2.613 ± 0.101<br>2.54<br>(2.45–2.765)	4.13 ± 0.13<br>4.11<br>(3.84–4.35)	3.130 ± 0.129<br>3.109<br>(2.836–3.350)	0.428 ± 0.038<br>0.453<br>(0.355–0.494)
WKY, Raspberry Seeds<br>(RBS)	3.25 ± 0.07<br>3.21<br>(3.10–3.41)	0.923 ± 0.033<br>0.940<br>(0.8425–0.9675)	1.968 ± 0.130<br>2.135<br>(1.583–2.203)	2.327 ± 0.054<br>2.325<br>(2.238–2.445)	3.53 ± 0.09<br>3.51<br>(3.34–3.78)	2.533 ± 0.091<br>2.505<br>(2.340–2.775)	0.325 ± 0.028<br>0.345<br>(0.249–0.379)
SHR, Control<br>(C)	2.17 ± 0.04<br>2.17<br>(2.06–2.27)	0.602 ± 0.021<br>0.585<br>(0.5650–0.6450)	1.595 ± 0.104<br>1.470<br>(1.403–1.910)	1.567 ± 0.037<br>1.555<br>(1.488–1.625)	3.62 ± 0.10<br>3.61<br>(3.40–3.84)	2.618 ± 0.105<br>2.609<br>(2.402–2.838)	0.420 ± 0.038<br>0.396<br>(0.345–0.528)
SHR, Raspberry Seeds<br>(RBS)	2.04 ± 0.03<br>2.03<br>(1.99–2.10)	0.632 ± 0.028<br>0.610<br>(0.5900–0.6775)	1.670 ± 0.092<br>1.665<br>(1.448–1.905)	1.440 ± 0.035<br>1.43<br>(1.37–1.515)	3.44 ± 0.09<br>3.51<br>(3.17–3.62)	2.438 ± 0.094<br>2.510<br>(2.168–2.615)	0.421 ± 0.027<br>0.394<br>(0.372–0.487)
p–value							
WKY, C vs. WKY, RBS	0.1838	0.0733	0.3447	**0.0173**	**0.0036**		**0.05**
SHR, C vs. SHR, RBS	0.5424	0.7914	0.9734	0.0625	0.2311		>0.9999
WKY, C vs. SHR, C	**<0.0001**	**<0.0001**	**0.0078**	**0.0022**	**0.0117**		0.9969
WKY, RBS vs. SHR, RBS	**<0.0001**	**<0.0001**	0.3628	**<0.0001**	0.4891		**0.0342**

[a] Nontraditional lipid profile was calculated as: non-HDL-C = TC minus HDL-C; TC/HDL; atherogenic index = non-HDL-C/HDL-C; AIP, log10(TG/HDL-C). Data are expressed either as means ± SEM or the median (with Q1 and Q3), of n = 6 rats: two-way ANOVA/Tukey's. Bold values indicate statistically significant differences ($p \leq 0.05$). Abbreviations: AI, atherogenic index; AIP, atherogenic index of plasma; HDL-C, high density cholesterol; TC, total cholesterol; TG, triglycerides.

Table S4. Blood plasma biochemical indices.

Experimental group	AST (U/L)	ALT (U/L)	Uric acid (mmol/L)	Urea (mmol/L)	CAT (U/mL)	SOD (U/mL)
WKY, Control (C)	89.32 ± 1.658<br>89.80<br>(85.55–92.93)	28.25 ± 1.186<br>27.35<br>(25.90–31.05)	21.67 ± 2.011<br>21.00<br>(17.00–26.50)	5.225 ± 0.215<br>5.250<br>(4.735–5.608)	1090 ± 53.70<br>1117<br>(944.5–1193)	28.01 ± 0.05<br>28.01<br>(27.88–28.14)
WKY, Raspberry Seeds (RBS)	78.30 ± 2.709<br>77.85<br>(73.00–85.53)	29.25 ± 1.136<br>29.35<br>(26.58–31.63)	18.5 ± 1.586<br>19.00<br>(14.75–22.00)	5.16 ± 0.158<br>5.235<br>(4.755–5.478)	948.4 ± 32.03<br>942.4<br>(891.0–1024)	28.35 ± 0.327<br>28.13<br>(27.88–28.96)
SHR, Control (C)	100.50 ± 1.791<br>100.40<br>(97.00–103.40)	32.60 ± 1.418<br>33.05<br>(30.00–34.88)	14.5 ± 1.384<br>14.00<br>(12.25–17.00)	5.738 ± 0.137<br>5.740<br>(5.413–6.010)	1065 ± 19.98<br>1091<br>(1016–1096)	27.99 ± 0.105<br>28.09<br>(27.78–28.19)
SHR, Raspberry Seeds (RBS)	88.33 ± 1.924<br>87.80<br>(83.90–93.70)	30.52 ± 1.326<br>29.65<br>(27.58–34.08)	11.83 ± 1.222<br>12.00<br>(8.75–15.00)	5.852 ± 0.324<br>5.985<br>(5.460–6.325)	994.5 ± 23.03<br>995.2<br>(948.6–1039)	28.42 ± 0.298<br>28.36<br>(27.87–28.80)
p–value						
WKY, C vs. WKY, RBS	**0.0095**	0.9259	0.2776	0.9975	**0.0468**	0.3095
SHR, C vs. SHR, RBS	**0.0045**	0.5888	0.4171	0.9873	**0.0390**	0.1797
WKY, C vs. SHR, C	**0.0087**	0.0738	**0.0035**	0.4692	0.6736	0.8182
WKY, RBS vs. SHR, RBS	**0.0183**	0.8632	**0.0062**	0.2304	0.2697	0.8701

Bold values indicate statistically significant differences ($p \leq 0.05$). Data are expressed either as means ± SEM or the median (with Q1 and Q3), of n = 6 rats: two-way ANOVA/Tukey's. Abbreviations: ALT, alanine aminotransferases; AST, aspartate aminotransferase; CAT, Catalase; SOD, Superoxide dismutase.
